# The effect of acute pain on risky and intertemporal choice

**DOI:** 10.1007/s10683-017-9515-6

**Published:** 2017-02-07

**Authors:** Lina Koppel, David Andersson, India Morrison, Kinga Posadzy, Daniel Västfjäll, Gustav Tinghög

**Affiliations:** 1 Center for Social and Affective Neuroscience, Department of Clinical and Experimental Medicine, Linköping University, Linköping, Sweden; 2 JEDI Lab, Division of Economics, Department of Management and Engineering, Linköping University, Linköping, Sweden; 3 Division of Psychology, Department of Behavioral Sciences and Learning, Linköping University, Linköping, Sweden; 4 Decision Research, Eugene, OR USA; 5 National Center for Priority Setting in Health Care, Department of Medical and Health Sciences, Linköping University, Linköping, Sweden

**Keywords:** Pain, Decision making, Dual-process theory, Risk, Intertemporal choice, C91, D81, D87, D90

## Abstract

Pain is a highly salient and attention-demanding experience that motivates people to act. We investigated the effect of pain on decision making by delivering acute thermal pain to participants’ forearm while they made risky and intertemporal choices involving money. Participants (n = 107) were more risk seeking under pain than in a no-pain control condition when decisions involved gains but not when they involved equivalent losses. Pain also resulted in greater preference for immediate (smaller) over future (larger) monetary rewards. We interpret these results as a motivation to offset the aversive, pain-induced state, where monetary rewards become more appealing under pain than under no pain and when delivered sooner rather than later. Our findings add to the long-standing debate regarding the role of intuition and reflection in decision making.

**Electronic supplementary material:**

The online version of this article (doi:10.1007/s10683-017-9515-6) contains supplementary material, which is available to authorized users.

## Introduction

How are our decisions affected in situations where we experience acute pain? The majority (70%) of Europeans report having one or more physical pain experiences per month (Vowles et al. 2014) and almost 20% report having chronic pain (Breivik et al. 2006). Over the past few years, the pain reliever Acetaminophen has remained the most or second most sold medication in the United States (Aitken et al. 2015). These data suggest that pain is a highly prevalent condition that potentially influences many everyday behaviors and decisions. In the present research, we investigated the effect of pain on risky and intertemporal choices involving money. Our findings have implications for understanding decision making in real life environments, where decisions are frequently made under non-optimal conditions.

Dual-process theories hold that decision making is based on an interaction between intuition (“system 1”) and reflection (“system 2”; Evans 2003; Evans and Stanovich 2013; Kahneman and Frederick 2002). Intuitive processes are typically characterized as fast, automatic, effortless, and emotional. Reflective processes are characterized as slow, controlled, effortful, and deliberative. In this study, we used pain as a means to temporarily inhibit the reflective system 2, thus making decisions more intuitive and system 1 based. Pain is a suitable manipulation of system 1/system 2 processing, because painful stimuli are highly salient and attention-demanding (Eccleston and Crombez 1999; Legrain et al. 2009). When in pain, reducing or eliminating the pain becomes first priority and other tasks receive less attention. Pain could thus be viewed as a form of cognitive load. Loewenstein (2000) refers to pain as a “hot” feeling state that causes people to “take extreme actions” (p. 429). The more intense the state, the greater the gap between what one feels compelled to do (system 1) and what one should do based on the consequences of one’s actions (system 2). The perception of pain also activates the insula, a brain region that is known to respond to monetary (“painful”) losses (Kuhnen and Knutson 2005; Paulus et al. 2003; Samanez-Larkin et al. 2007; Wu et al. 2012).

Research on chronic pain and decision making has mostly used the Iowa Gambling Task (Bechara et al. 1994), an emotional learning task in which players pick cards from decks of varying gain/loss ratios. Advantageous decks contain cards with small gains and small losses. Disadvantageous decks contain larger gains but also larger losses. Healthy subjects normally begin by sampling cards from each deck and then learn to stick to the advantageous, low-risk decks and to avoid the disadvantageous, high-risk ones. Patients with chronic pain keep choosing cards from both kinds of decks and therefore end up with less money than controls (Apkarian et al. 2004; Biagianti et al. 2012; Tamburin et al. 2014; Verdejo-García et al. 2009; Walteros et al. 2011). This learning impairment seems to be due to a lack of somatic markers, because chronic pain patients’ physiological arousal does not increase when they pick cards from disadvantageous decks, whereas it does in healthy controls (Elvemo et al. 2014). Patients with chronic pain are also more risk taking on tasks that do not involve a learning component, especially when high potential gains (as opposed to losses) are at stake (Berger et al. 2014).

The prediction that follows from research on chronic pain is that acute pain, like chronic pain, will increase risk taking overall. This prediction is somewhat in contrast with dual-process theories, which predict that inhibiting system 2 increases risk taking in the loss domain only, whereas it reduces risk taking in the gain domain. In a study by Porcelli and Delgado (2009), participants immersed their dominant hand in ice-cold water for 2 min, a procedure known as the cold-pressor task, before completing a decision-making task in which they chose between either two potential losses or two potential gains. The cold-pressor task is known to induce stress, which should lead to more system 1 processing. Indeed, participants who underwent the cold-pressor task were less risk taking in the gain domain but (marginally) more risk taking for equivalent gambles in the loss domain. Similar results have also been observed with time pressure (Kirchler et al. in press). An important distinction between the present study and the one by Porcelli and Delgado (2009) is that their participants experienced the painful stimulus before, rather than during, the decision phase. Thus, the authors explain their results in terms of stress rather than ongoing pain. In the present study, we investigated decisions made during the experience of a painful stimulus. On the one hand, we believe that our manipulation inhibits the reflective system 2 more effectively than common manipulations such as cognitive load, time pressure, and stress. On the other hand, pain has a unique neural signature (Wager et al. 2013), implying that its effect on behavior and decision making might differ from the effect of other types of hot system 1 states.

Another prediction from dual-process theories is that inhibition of system 2 leads to a greater preference for immediate over future rewards, a phenomenon known as temporal discounting. This prediction is supported by research showing that the evolutionarily old limbic and paralimbic systems are activated when participants choose immediate rewards, whereas evolutionarily newer prefrontal areas are activated when they choose delayed rewards (McClure et al. 2004). Moreover, cognitive load has been shown to lead to greater discounting of delayed monetary rewards (Hinson et al. 2003), and heroin addicts in the “hot” state of drug craving not only show a greater preference for immediate over delayed delivery of heroin but also for immediate over delayed delivery of monetary rewards (Giordano et al. 2002). Given that pain could be viewed as a hot feeling state that reduces self-control (Loewenstein 1996, 2000), participants in pain should show greater preference for immediate over future rewards, even if those rewards are not directly related to the pain. This prediction is in line with research showing that participants who report greater anticipation of pain prior to painful experiences (which is linked to pain sensitivity; Brañas-Garza et al. 2010) are more impatient for monetary rewards (Brañas-Garza et al. 2012).

In the present study, pain was delivered at participants’ subjective pain threshold while they made dichotomous decisions with real monetary consequences. We explored the effect of pain on risky choice (both gains and losses) and intertemporal choice. Participants completed the decision-making tasks both with and without pain (in counterbalanced order), which allowed us to explore the effect of pain both between and within subjects.

## Method

### Participants and materials

109 participants (35% female; *M* age = 23.4 years, *SD* = 3.5) were recruited using the Online Recruitment System for Economic Experiments (ORSEE; Greiner 2015) at Linköping University, Sweden. A sample-size calculation based on means and standard deviations from a previous study from our lab (Kirchler et al. in press) and with 70% power showed that 50 participants were needed in each condition. Data collection continued until all scheduled experimental sessions for the week of the 100th participant had been completed. All participants gave their informed consent prior to participation. Individuals were not allowed to participate if they were taking anxiolytic, antidepressive, or pain relieving medication. Participants were paid 100 SEK (approx. 12 USD) as a show-up fee plus or minus the amount from one randomly selected decision. Delayed payoffs from the intertemporal choice task were paid using Swish, a free, popular smartphone app that facilitates immediate money transfers between bank accounts. Participants who had not already installed Swish on their phone were required to do so before participating in the study. Two male participants were excluded due to technical problems during the experiment, leaving 107 participants in the final sample.

Painful heat stimulation was delivered to the distal part of participants’ left dorsal forearm using a 3 × 3 cm Thermal Stimulator Probe (Q-sense, Medoc). Prior to the experiment, participants’ pain threshold for heat was determined by a procedure following Perini et al. (2013), in which the thermode had a baseline temperature of 32 °C and increased at a speed of 1 °C/s. Participants’ task was to press a mouse button positioned in their right hand when the stimulation reached the border between painful and too painful. For safety reasons, the temperature never exceeded 50 °C. After they had pressed, the temperature returned to baseline. The procedure was repeated four times. The highest achieved temperature was selected as the pain threshold (max. 49 °C). Participants then completed a 1 min trial block in which the temperature varied between their pain threshold and 2 °C below the threshold, just as it would during the experimental trials (see below). Participants were told that the stimulation was meant to be painful but endurable and were allowed adjust to a lower or higher temperature if they perceived the stimulation as too painful or not painful enough, respectively. Pain thresholds after adjustments ranged between 40 °C and 49 °C (*M* = 48.07, *SD* = 1.38). Fifty-four participants had the maximum pain threshold of 49 °C. Pain thresholds did not differ as a function of the order in which participants were in the pain and control conditions, *t*(105) = .80, *p* = .425. A manipulation check was conducted on the last 56 participants, who indicated how painful they perceived the stimulation on a scale from 1 (*not at all painful*) to 10 (*extremely painful*). This confirmed that the stimulation was perceived as very painful (*M* = 8.43, *SD* = 1.36) and that the subjective experience of the stimulation did not differ as a function of the order of conditions, *t*(54) = .56, *p* = .578.

### Experimental design

We used a crossover design in which participants performed three decision-making tasks twice: once with pain and once without pain. Participants thus served as their own controls. The order of the tasks was the same for all participants, but the order of the pain and control conditions was randomized between participants so that about half the participants (n = 57, 32% female) were in the pain condition first and the other half (n = 50, 40% female) were in the control condition first. In the pain condition, painful heat stimulation as described above was delivered to participants’ left forearm continuously for 60 s while they completed each task. In the control condition, the thermode was placed on participants’ forearm but the temperature remained at baseline (32 °C). Everything else was identical between conditions. Each task lasted for 60 s and was followed by a break of at least 30 s during which the thermode was removed from participants’ arm. Thus, pain was only delivered during the decision phase. General instructions were given before the experiment started and task-specific instructions were given before each task. Participants were informed that one of their decisions would be randomly selected for actual payment. The tasks were presented on a computer screen and were programmed in Qualtrics. A translation of the complete instructions for the experiment is provided in the Supplemental Materials.

#### Risky gains task

On each of five trials, participants chose between a safe option (receiving a sum of money with certainty) and a risky option (receiving a sum of money with 50% probability). Participants had 12 s to respond on each trial and could not proceed to the next trial until the given time had elapsed. This was done to minimize potential confounding effects of time pressure and response times.

#### Risky losses task

On each of five trials, participants chose between a safe option (losing a sum of money with certainty) and a risky option (losing a sum of money with 50% probability). Participants again had 12 s to respond on each trial.

#### Intertemporal choice task

On each of four^1^ trials, participants chose between receiving an immediate (smaller) monetary reward and a delayed (larger) monetary reward. The immediate reward was delivered either on the same day or the day after the experiment and the delayed reward was delivered one, two, five, or six days following the experiment. Participants had 10 s to respond on each trial.

### Data analysis

We first performed paired samples t-tests to investigate whether the proportion of risky and impatient choices, compared to the total number of trials in each task, was greater in the pain condition than in the control condition. We then performed regression analyses in order to confirm the results from the t-tests while also controlling for age and gender. Our regression model for the risky choice tasks was specified as follows:

1 $$y_{ik} = \beta_{0} + \beta_{1} Pain + \beta_{2} Round + \beta_{3} Ratio + \beta_{4} X_{i} + \in_{ik}$$

where the dependent variable $y_{ik}$ is a dummy variable indicating whether participant *i* chose the risky option in trial *k*. *Pain* is a dummy for the pain condition and *Round* is a dummy for the *second* round of the tasks, i.e. the second time participants performed the tasks. An alternative model also included the interaction term *Pain* × *Round*, which allows the effect of pain to differ across the two task rounds. *Ratio* is the ratio between the expected value of the risky option and the expected value of the safe option on each trial (standardized). **X**_*i*_ is the control variables age and gender. The model was estimated using OLS and standard errors were corrected for clustering on the individual level.

Our regression model for the intertemporal choice task was identical to the model above except the dependent variable $y_{ik}$ was a dummy variable indicating whether participant *i* chose the immediate option in trial *k* and *Ratio* was replaced with *Delay*, which denotes the difference in days (one or five) between the immediate and the delayed reward. Thus, the regression model for the intertemporal choice task was specified as follows:

2 $$y_{ik} = \beta_{0} + \beta_{1} Pain + \beta_{2} Round + \beta_{3} Delay + \beta_{4} X_{i} + \in_{ik}$$

For all choice tasks, we also investigated whether the effect of pain differed between male and female participants. To do this, we performed regression analyses using the models specified above where we included the interaction term *Pain* × *Female*, which allows the effect of pain to differ across genders.

We also estimated risk aversion and discounting parameters for the pain and control conditions. For the estimation of risk aversion, we assumed constant relative risk aversion (CRRA) and the following utility function:

3 $$ u\left( x \right) = \left\{ {\begin{array}{*{20}c} {\frac{{x^{1 - r} }}{1 - r}\,   \,             {\hbox{if}}\,\, x \ge \,0} \\ { - \frac{{\left( { - x} \right)^{1 - r} }}{1 - r}\,\,                {\hbox{if}}\,\, x \,< \,0} \\ \end{array} } \right. $$

where *r* is the coefficient of relative risk aversion and *x* is the monetary outcome. With this parameterization, *r* = 0 denotes risk-neutral behavior, *r* > 0 denotes risk aversion (risk-seeking behavior), and *r* < 0 denotes risk-seeking behavior (risk aversion) for gains (losses). Using this utility function, we denoted the expected utility of each alternative (A) as:

4 $$EU\left( A \right) = \mathop \sum \limits_{x \in A} p\left( x \right)u\left( x \right)$$

We calculated the difference in expected utility between the safe option (S) and the risky option (R) using:

5 $$\Delta EU = EU\left( S \right) - EU\left( R \right)$$

The likelihood function is:

6 $$ L = \left\{ {\begin{array}{ll} {\varPhi \left( {\Delta EU} \right) }&\quad {\hbox{if}}\, \hbox{Safe} \\ { 1 - \varPhi \left( {\Delta EU} \right)} &\quad {\hbox{if}}\, \hbox{Risky} \\ \end{array} } \right. $$

where Φ is the cumulative distribution function of the standard normal distribution. The likelihood function was estimated using maximum likelihood with the Broyden-Fletcher-Goldfarb-Shanno algorithm. We estimated the average risk factor for the whole sample in the pain and control conditions in the following way (allowing for heterogeneity in risk preferences):

7 $$\widehat{r} = r_{0} + \alpha_{0} Pain + \alpha_{1} Round$$

where $r_{0}$ represents the estimate of the constant, *Pain* is a dummy for the pain condition, and *Round* is a dummy for the second round of the task. An alternative model also included the interaction term *Pain* × *Round*, which allows the effect of pain to differ across the two task rounds. Standard errors were clustered at the individual level. Estimates were provided separately for gains and losses in order to allow for different curvature of the utility function for positive and negative outcomes.

For the discount factor, we assume exponential discounting and the following function for discounted utility:

8 $$u\left( {\text{x}} \right) =\updelta^{t} u_{t} \left( {\text{x}} \right)$$

where $\delta$ denotes the discount factor and *t* denotes the number of days until the outcome will be realized. In function (8) we assume that the utility function at time t, $u_{t} \left( x \right)$, takes form as in (3) with estimated average risk factor as described above. Using (8) we calculated discounted utility separately for the immediate and the delayed reward. We then calculated the difference in discounted utility between the delayed (D) and the early (E) options using:

9 $$\Delta U = U\left( D \right) - U\left( E \right)$$

We can then describe the likelihood function as:

10 $$ L = \left\{ {\begin{array}{ll} \varPhi \left( {\Delta U} \right)  &\quad \hbox{if}\quad \hbox{Delayed} \\  1 - \varPhi \left( {\Delta U} \right)  & \quad      \hbox{if}\quad \hbox{Early} \\ \end{array} } \right. $$

where Φ is the cumulative distribution function of the standard normal distribution. We used the same estimation method as for risk preferences. Similarly, we estimated the average discount factor for the whole sample in the pain and control conditions, allowing for heterogeneity in time preferences:

11 $$\widehat{\delta } = \delta_{0} + \alpha_{0} Pain + \alpha_{1} Round$$

where $\delta_{0}$ represents the estimate of the constant, *Pain* is a dummy for the pain condition, and *Round* is a dummy for the second round of the task. An alternative model also included the interaction term *Pain* × *Round*, which allows the effect of pain to differ across the two task rounds. Standard errors were clustered at the individual level.

We finally considered data at the individual level by investigating whether pain had the same effect on all individuals in each task and whether individuals who changed their behavior in response to pain on one task also changed their behavior in a similar fashion on the other tasks. For the latter, we calculated the difference in the proportion of risky and impatient choices in the pain condition compared to the control condition, for each individual and task. We then performed Pearson correlations of the difference scores to explore the relationship between choices in the three tasks.

## Results

### The effect of pain on risky choice

**Fig. 1:**
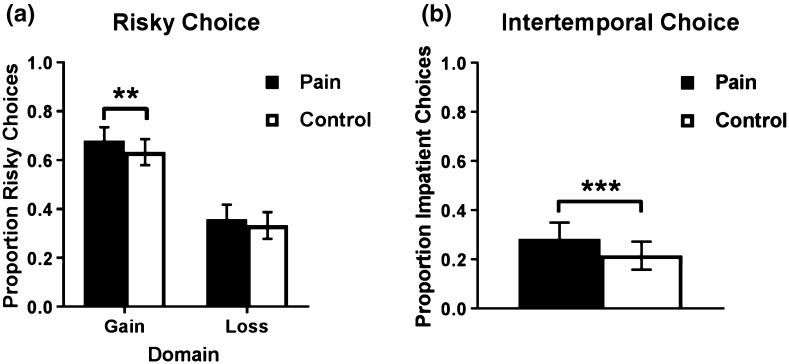
Proportion of participants’ **a** risky and **b** impatient choices as a function of condition (pain vs. control), with *error bars* showing 95% confidence intervals. *p < .10, **p < .05, ***p < .01

Figure 1a displays the proportion of participants’ risky choices compared to the total number of trials in each condition (pain vs. control) and domain (gain vs. loss; for trial-by-trial results, see Table S1 in the Supplemental Material). A paired samples *t* test showed that the proportion of risky choices in the gain domain was greater in the pain condition than in the control condition, *M*_*pain*_ = .68 (95% CI [.63, .74]), *M*_*control*_ = .63 (95% CI [.58, .69]), *t*(106) = 2.06, *p* = .042, *d* = .12. The regression analyses confirm this finding (see Table 1). That is, participants were more likely to choose the risky option in the pain condition than in the control condition, *β* = .049, *p* = .035. Age, gender, and *Round* (i.e., whether it was the first or second time participants performed the task) had no significant effect on decisions. However, there was a significant interaction between *Pain* and *Round*, indicating that participants who were in the pain condition first were on average more risk-seeking than participants who were in the control condition first. We return to this interaction later. There was no evidence that the effect of pain differed between the genders (see Table S2 in the Supplemental Material). The estimated parameter of risk aversion in the gain domain, when controlling for *Round*, was .133 in the pain condition and .146 in the control condition. This difference was not statistically significant, *p* = .348 (see Table S3 in the Supplemental Material). A paired samples *t* test found no difference in response times between the pain and control conditions, *M*_*pain*_ = 4.23 s (95% CI [3.93, 4.53]), *M*_*control*_ = 4.32 s (95% CI [4.05, 4.58]), *t*(106) = –.53, *p* = .599, *d* = –.05.

**Table 1:** Regression analyses of risky and intertemporal choices

	Risky gains		Risky losses		Intertemporal choice	
	(1)	(2)	(1)	(2)	(1)	(2)
Pain	.049**	.184***	.021	.072	.071***	.084
	(.023)	(.055)	(.025)	(.056)	(.018)	(.058)
Round	.011	.146***	.028	.079	.034*	.047
	(.023)	(.054)	(.025)	(.052)	(.018)	(.058)
Pain × round		–.270***		–.101		–.027
		(.099)		(.099)		(.116)
Female	–.078	–.064	.116**	.121**	–.033	–.032
	(.049)	(.051)	(.049)	(.049)	(.056)	(.057)
Age	.003	.002	.003	.002	–.006	–.006
	(.008)	(.008)	(.010)	(.009)	(.007)	(.007)
Ratio	.202***	.202***	–.194***	–.194***		
	(.014)	(.014)	(.016)	(.016)		
Delay					.076***	.076***
					(.009)	(.009)
Constant	.577***	.536***	.207	.192	.122	.118
	(.193)	(.193)	(.227)	(.226)	(.167)	(.170)

This table reports OLS coefficient estimates (robust standard errors corrected for clustering on the individual level in parentheses). For the risky choice tasks, the dependent variable is a dummy variable indicating whether participants chose the risky option. For the intertemporal choice task, the dependent variable is a dummy variable indicating whether they chose the immediate reward. “Pain” is a dummy for the pain condition. “Round” is a dummy for the *second* round of the tasks, i.e. the second time the participants performed the tasks. “Pain × Round” is the interaction between the pain condition and the task round, allowing the effect of pain to differ across the two task rounds. “Female” is a gender dummy. “Age” is the participant’s age in years. “Ratio” is the ratio between the expected value of the risky option and the expected value of the safe option on each trial (standardized). “Delay” is the difference in days (1 or 5) between the immediate and the delayed reward in the intertemporal choice task

* *p* < .10, ** *p* < .05, *** *p* < .01

In the loss domain, there was no statistically significant difference in the proportion of risky choices between the two conditions, *M*_*pain*_ = .36 (95% CI [.30, .42]), *M*_*control*_ = .33 (95% CI [.28, 39]), *t*(106) = 1.00, *p* = .322, *d* = .07. The regression analyses did not find a significant effect either (see Table 1). That is, participants were not more likely to choose the risky option in the pain condition than in the control condition, *β* = .021, *p* = .400. There was, however, a significant effect of gender, such that women were more likely than men to choose the risky option, *β* = .116, *p* = .020. Age and *Round* had no significant effect on decisions and there was no significant interaction between pain and *Round* or between pain and gender (see Table S3 in the Supplemental Material). The estimated parameter of risk aversion in the loss domain, after controlling for *Round*, was .136 in the pain condition and .137 in the control condition. This difference was not statistically significant, *p* = .976 (see Table S3 in the Supplemental Material). There was no difference in response times between the pain and control conditions, *M*_*pain*_ = 4.48 s (95% CI [4.19, 4.76]), *M*_*control*_ = 4.58 s (95% CI [4.27, 4.89]), *t*(106) = –.57, *p* = .569, *d* = –.06.

**Fig. 2:**
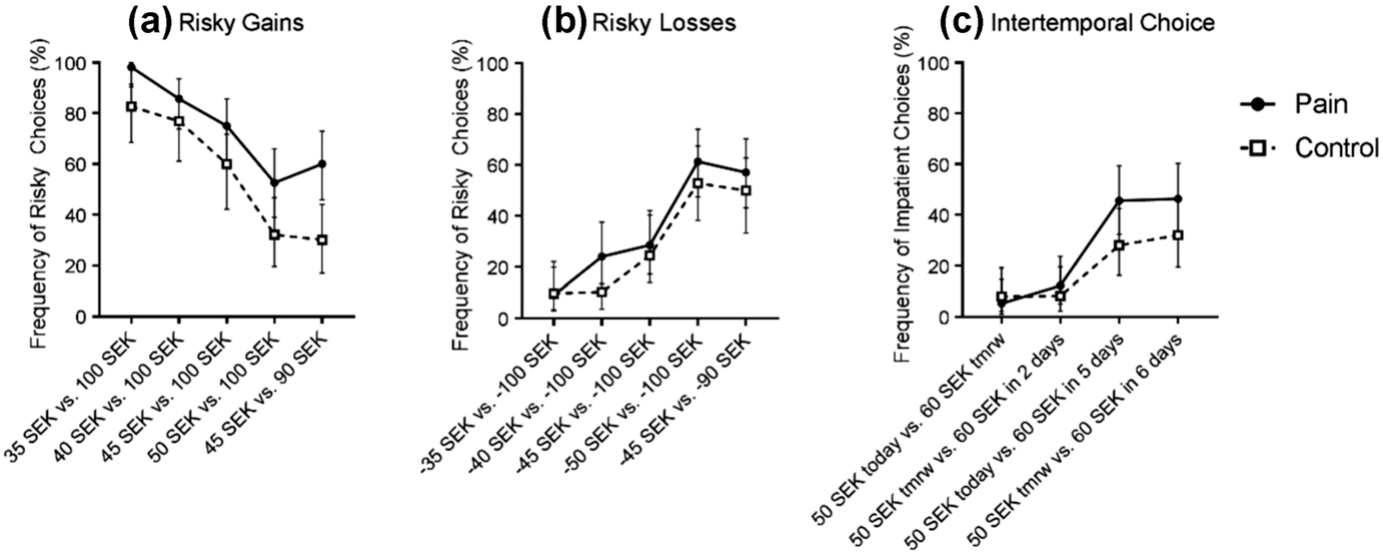
Percent frequency of **a** risky choices in the gain domain, **b** risky choices in the loss domain, and **c** impatient choices in the intertemporal choice task, presented per trial as a function of condition (pain vs. control) for the first round of each task, with error bars showing 95% confidence intervals

In short, the results from the risky choice tasks suggest that acute pain increases risk seeking in the gain domain, but not in the loss domain. However, there was also a significant interaction between pain and the order in which participants were in the pain and control conditions—that is, in the gain domain, participants who were in the pain condition first were always more risk seeking than participants who were in the control condition first. An additional analysis examined only the first round of each task (i.e., the first time participants completed each task), where choices were not influenced by choices from previous rounds. Results are presented separately for each trial in Fig. 2 (see also Table S4 in the Supplemental Material). An independent samples t-test showed that the overall proportion of risky choices in the gain domain was significantly greater in the pain condition than in the control condition, *M*_*pain*_ = .74 (95% CI [.67, .81]), *M*_*control*_ = .55 (95% CI [.47, .64]), *t*(105) = 3.47, *p* < .001, *d* = .69. The difference in the proportion of risky choices in the loss domain was not statistically significant, *M*_*pain*_ = .37 (95% CI [.28, .45]), *M*_*control*_ = .30 (95% CI [.22, .38]), *t*(105) = 1.09, *p* = .280, *d* = .23. The regression analyses corroborate these findings (see Table 1). That is, pain increased the likelihood of choosing the risky option in the gain domain, *β* = .184, *p* = .001, but not in the loss domain, *β* = .072, *p* = .160. In the gain domain, the estimated parameter of risk aversion was .088 in the pain condition and .190 in the control condition. This difference was statistically significant, *p* < .001. In the loss domain, the estimated parameter of risk aversion was .146 in the pain condition and .124 in the control condition. This difference was not statistically significant, *p* = .452 (see Table S3 in the Supplemental Material). Thus, the between-subjects results confirm the finding that pain increases risk seeking for gains but not for losses.

### The effect of pain on intertemporal choice

Figure 1b shows the proportion of participants’ impatient choices in the pain vs. control condition compared to the total number of trials in the intertemporal choice task (for trial-by-trial results, see Table S1 in the Supplemental Material). A paired samples t-test showed that the proportion of impatient choices was greater in the pain condition than in the control condition, *M*_*pain*_ = .28 (95% CI [.22, .35]), *M*_*control*_ = .22 (95% CI [.22, .27]), *t*(106) = 3.84, *p* = .0002, *d* = .16. The results from the regression analyses corroborate this finding (see Table 1). That is, participants were more likely to choose the impatient option in the pain condition than in the control condition, *β* = .071, *p* = < .001. Age, gender, and *Round* had no significant effect on the proportion of impatient choices and there was no interaction between pain and *Round* or between pain and gender (see Table S2 in the Supplemental Material). The estimated discount factor, after controlling for *Round*, was .969 in the pain condition and .971 in the control condition. This difference was statistically significant, *p* < .001. There was no difference in response times between the pain and control conditions, *M*_*pain*_ = 3.62 s (95% CI [3.38, 3.48]), *M*_*control*_ = 3.71 s (95% CI [3.48, 3.93]), *t*(106) = –.58, *p* = .566, *d* = –.06.

Results from the first round of the intertemporal choice tasks are presented in Fig. 2c (see also Table S4 in Supplemental Material). The difference in the proportion of impatient choices was in the same direction as in the above analyses but was not statistically significant, *M*_*pain*_ = .27 (95% CI [.19, .35]), *M*_*control*_ = .19 (95% CI [.11, .27]), *t*(105) = 1.46, *p* = .147, *d* = .27. The regression analyses did not yield a significant effect either, *β* = .084, *p* = .144, (see Table 1). The estimated discount factor was .969 in the pain condition and .971 in the control condition. This difference was not statistically significant, *p* = .119 (see Table S3 in the Supplemental Material). However, the non-significance might just reflect the decrease in statistical power when investigating between-subjects differences only. In contrast to the risky choice tasks, the order of conditions did not significantly influence the effect of pain on decision making in the intertemporal choice task. Thus, we conclude that pain increases preferences for immediate over future rewards.

### Individual differences in the effect of pain across tasks

We found no evidence that the same individuals were driving the effect across all tasks, because the pain vs. control difference scores for the three tasks were only weakly correlated at best (gain and loss, *r* = −.181, *p* = .063; gain and intertemporal choice, *r* = −.071, *p* = .467; loss and intertemporal choice, *r* = .175, *p* = .074). Thus, we conclude that there was not just one subgroup of participants whose decisions were influenced by the painful stimulation. See Figure S1 in the Supplemental Material for a graphical depiction of the effect of pain on each task at the individual level.

## Discussion

Pain is a highly aversive and attention-demanding experience (Eccleston and Crombez 1999; Legrain et al. 2009). Although responses to pain have evolutionarily adaptive value, performance on cognitively demanding tasks such as decision making may be compromised. Here we showed that acute physical pain influences risky and intertemporal choices involving money. Participants experiencing a painful stimulus were more risk seeking for gains but not for losses and showed greater preferences for immediate (smaller) over future (larger) monetary rewards than participants experiencing a non-painful stimulus. We interpret these results as a motivation to compensate for the negative state induced by pain.

The results indicate that pain increases risk seeking for monetary gains but not for monetary losses. Although in line with previous research on chronic pain patients (Apkarian et al. 2004; Berger et al. 2014; Biagianti et al. 2012; Elvemo et al. 2014; Tamburin et al. 2014; Verdejo-García et al. 2009; Walteros et al. 2011), these findings partly go against dual-process theories, which predict that inhibition of system 2 leads to greater reliance on automatic biases such as the reflection effect of prospect theory (Evans 2003; Evans and Stanovich 2013; Kahneman and Frederick 2002; Kirchler et al. in press; Porcelli and Delgado 2009). Thus, our findings suggest that pain does more than inhibit the reflective system 2—it produces risk seeking behavior for potential rewards. This suggestion is supported by previous research showing that pain increases the motivation to obtain a monetary reward, as indicated by faster response times in the monetary incentive delay (MID) task during pain than during a control condition (Gandhi et al. 2013). Seeking risky rewards may even be an adaptive response to pain, because monetary gains can reduce the subjective intensity and unpleasantness of a painful experience (Becker et al. 2013). Thus, a risky choice in the present study could be interpreted as an attempt to relieve pain.

Pain also increased preferences for immediate (smaller) over future (larger) rewards. These results are in line both with dual-process approaches to decision making and with previous evidence that monetary rewards can offset the pain-induced negative state (Becker et al. 2013). If monetary rewards act as pain relievers, and pain is experienced temporarily at the moment the decision is made, then it makes sense to choose an immediate over a delayed reward. After all, a pain reliever is most useful in a moment of pain. These findings have implications for understanding how “hot” feeling states influence decision making. Feeling states such as pain have previously been predicted to only influence decisions that are directly related to those states (Loewenstein 1996). For example, drug addicts temporally discount drugs to a greater extent than other kinds of rewards (Giordano et al. 2002). However, the current study shows that the hot state of pain influences temporal discounting of money, a reward that is not directly related to pain.

Our general findings that pain influences risk seeking and impatience can be linked to a literature on the role of incidental emotions in decision making. Raghunathan and Pham (1999) noted that sad participants were more risk seeking whereas anxious participants were less risk seeking than control participants and suggested that emotions may have different effects on judgments and decisions depending on their informational content and implicitly activated goals. Feelings of sadness can be interpreted as a lack of something rewarding, which activates the goal of reward-seeking. Feelings of fear or anxiety involve uncertainty and lack of control, which activate the goal of uncertainty reduction. In line with this “mood repair” account, anticipation of painful, as opposed to non-painful, electric shocks has been found to reduce risk taking in an investment task (Cohn et al. 2015). Furthermore, activity in the ventromedial prefrontal cortex and ventral striatum predict choices in a risk task in the absence of threat, whereas activity in the insula predicts choices in the presence of threat (Engelmann et al. 2015). It should be noted that these studies involve anticipation of painful shocks, which are short-lived and induce a state of anxiety and uncertainty. In contrast, the present study involved continuous delivery of thermal stimulation, which induces an ongoing state of pain but which lacks the uncertainty component. This difference between ongoing, actual pain and anticipation of painful shock might explain why we observed an effect of experienced pain on choices but Cohn et al. (2015) did not. Furthermore, a speculative interpretation of our findings that is in line with the mood repair literature is that pain induces a negative emotional state and that participants attempt to repair their mood by opting for risker but higher reward options or rewards that are delivered closer in time.

A limitation of the present study is that the choice tasks were presented in the same order for all participants (i.e., risky gains, risky losses, intertemporal choice). We therefore cannot rule out the possibility of order effects. For instance, the reason we did not find a significant effect of pain in the loss domain could be that participants habituated to the pain and were more familiar with the type of choice task. However, we did observe an effect in the intertemporal choice task even though it was placed last in the experiment. Moreover, Berger et al. (2014) found that patients with chronic pain were more risk seeking than control participants, but only for gains and not for losses, which is in line with our findings. A second limitation is the possibility that only individuals with a high tolerance for pain signed up to participate. It is unclear how such self-selection bias may have influenced our results. If we were to speculate, it seems most likely that the effect of pain is stronger among individuals who did not sign up, because anticipation of pain has been shown to be positively correlated with both sensitivity to pain (Brañas-Garza et al. 2010) and impatience for monetary rewards (Brañas-Garza et al. 2012).

Future research needs to identify the exact processes behind the effects observed in the present study. For instance, does pain influence the value function or the probability weighting function of prospect theory? Furthermore, in the present study, we compared the influence of “hot” (painful) and “cold” (non-painful) states on decision-making while keeping the decisions constant. However, pain can also result from the decision itself, as when an individual experiences a monetary loss (Kuhnen and Knutson 2005; Paulus et al. 2003; Samanez-Larkin et al. 2007; Västfjäll et al. 2016; Wu et al. 2012). Probability weighting for painful electric shocks has been found to be similar to probability weighting for monetary losses (Berns et al. 2007), Future research should investigate the relative contributions of incidental pain, which is unrelated to the decision at hand, and integral pain, which results from the decision itself. Additionally, experiences such as social rejection (Eisenberger et al. 2003) and economic insecurity (Chou et al. 2016) can feel physically painful. The question remains whether the effect of pain on risky and intertemporal choice generalizes to pain from non-physical sources. Finally, it would be good if future replication studies were conducted, especially for risky choices given that our results were less conclusive for this domain when modeled within subject.

In conclusion, the present study demonstrates a behavioral effect of acute, physical pain on economic decision making. Participants were more risk seeking (for gains, but not for losses) and more impatient when experiencing a painful, compared to a non-painful, stimulus. It is possible that the effect is even greater in real-life settings outside the lab, where individuals have little to no control over the pain they experience and where they may be less aware that the pain influences their behavior and decisions. Thus, our findings contribute to the understanding of decision making in everyday life, which is filled with painful experiences.

## Electronic supplementary material

Below is the link to the electronic supplementary material.

Supplementary material 1 (DOCX 217 kb)

## Footnotes

1 We originally included a fifth question (20 s) in which participants indicated how much a sum of money (1000 SEK in the first round and 100 SEK in the second) would be worth if given to them a month from now. However, because 27% of participants failed to respond on time in the first round of the tasks, we have omitted that question from our data analysis.
