# Supplementary material for: The effect of acute pain on risky and intertemporal choice
Source: Exp Econ. 2017 Feb 7;20(4):878–93. doi: 10.1007/s10683-017-9515-6 (PMC5665967; doi:10.1007/s10683-017-9515-6)
Supplement: Supplementary file 1 — Supplementary material 1 (DOCX 217 kb) [file 10683_2017_9515_MOESM1_ESM.docx]

**Supplemental Material**

**Table S1** Estimates of risk aversion (*r*) and discounting (δ) parameters

|  | Risk aversion for gains (*r^+^*) | |  | Risk aversion for losses (*r*^–^) | |  | Discount factor (δ) | |
| --- | --- | --- | --- | --- | --- | --- | --- | --- |
|  | (1) | (2) |  | (1) | (2) |  | (1) | (2) |
| Pain | –.013  (.014) | –.102***  (.026) |  | .000  (.015) | .022  (.029) |  | –.002***  (.000) | –.002  (.002) |
| Round | –.017  (.014) | –.116***  (.026) |  | .003  (.015) | .027  (.029) |  | –.001*  (.000) | –.001  (.002) |
| Pain × Round |  | .182***  (.049) |  |  | –.048  (.052) |  |  | .001  (.003) |
| Constant | .146***  (.015) | .190***  (.018) |  | .137***  (.019) | .124***  (.022) |  | .971***  (.001) | .971***  (.001) |

*Notes.* This table reports coefficient estimates of risk aversion and discounting (robust standard errors corrected for clustering on the individual level in parentheses). “Pain” is a dummy for the pain condition. “Round” is a dummy for the *second* round of the tasks, i.e. the second time the participants performed the tasks. “Pain × Round” is the interaction between the pain condition and the task round, allowing the effect of pain to differ across the two task rounds.

* *p* < .10, ** *p* < .05, *** *p* < .01

**Table S2** Frequency (percent) of risky and impatient choices per trial in each task, as a function of condition (pain vs. control)

|  |  | Condition | |  | McNemar test |
| --- | --- | --- | --- | --- | --- |
|  |  | Pain | Control |  | *p* |
| Risky gains | |  |  |  |  |
|  | 35 SEK vs. 100 SEK | 101 (95.3%) | 97 (91.5%) |  | .157 |
|  | 40 SEK vs. 100 SEK | 84 (80.0%) | 88 (83.8%) |  | .317 |
|  | 45 SEK vs. 100 SEK | 71 (71.0%) | 73 (73.0%) |  | .695 |
|  | 50 SEK vs. 100 SEK | 46 (43.0%) | 39 (36.5%) |  | .209 |
|  | 45 SEK vs. 90 SEK | 54 (52.4%) | 35 (34.0%) |  | .001 |
| Risky losses | |  |  |  |  |
|  | -35 SEK vs. -100 SEK | 8 (7.8%) | 11 (10.7%) |  | .317 |
|  | -40 SEK vs. -100 SEK | 17 (17.2%) | 15 (15.2%) |  | .655 |
|  | -45 SEK vs. -100 SEK | 30 (29.7%) | 26 (25.7%) |  | .450 |
|  | -50 SEK vs. -100 SEK | 63 (61.8%) | 62 (60.8%) |  | .835 |
|  | -45 SEK vs. -90 SEK | 59 (57.3%) | 55 (53.4%) |  | .480 |
| Intertemporal choice | |  |  |  |  |
|  | 50 SEK today vs. 60 SEK tomorrow | 12 (11.2%) | 7 (6.5%) |  | .096 |
|  | 50 SEK today vs. 60 in two days | 14 (13.1%) | 9 (8.5%) |  | .096 |
|  | 50 SEK tomorrow vs. 60 SEK in five days | 47 (43.9%) | 36 (33.6%) |  | .008 |
|  | 50 SEK tomorrow vs. 60 SEK in six days | 48 (45.3%) | 40 (37.4%) |  | .021 |
|  |  |  |  |  |  |

**Table S3** Regression analyses of risky and intertemporal choices, including interaction between pain and gender

|  | Risky gains | |  | Risky losses | |  | | Intertemporal choice | |
| --- | --- | --- | --- | --- | --- | --- | --- | --- | --- |
|  | (1) | (2) |  | (1) | (2) |  | (1) | | (2) |
| Pain | 0.052* | 0.187*** |  | 0.012 | 0.063 |  | 0.051** | | 0.065 |
|  | (.027) | (.056) |  | (.033) | (.058) |  | (.020) | | (.059) |
| Round | 0.011 | 0.146*** |  | 0.027 | 0.078 |  | 0.032* | | 0.045 |
|  | (.023) | (.056) |  | (.025) | (.052) |  | (.018) | | (.058) |
| Pain × Round |  | –0.270*** |  |  | –0.101 |  |  | | –0.027 |
|  |  | (.099) |  |  | (.099) |  |  | | (.116) |
| Female | –0.074 | –0.060 |  | 0.103** | 0.108** |  | –0.061 | | –0.059 |
|  | (.054) | (.054) |  | (.051) | (.051) |  | (.056) | | (.057) |
| Pain × Female | –0.009 | –0.008 |  | 0.025 | 0.026 |  | 0.055 | | 0.055 |
|  | (.050) | (.050) |  | (.052) | (.052) |  | (.039) | | (.039) |
| Age | 0.003 | 0.002 |  | 0.003 | 0.002 |  | –0.006 | | –0.006 |
|  | (.008) | (.008) |  | (.010) | (.009) |  | (.007) | | (.007) |
| Ratio | 0.202*** | 0.202*** |  | –0.194*** | -0.194*** |  |  | |  |
|  | (.014) | (.014) |  | (.016) | (.016) |  |  | |  |
| Delay |  |  |  |  |  |  | 0.076*** | | 0.076*** |
|  |  |  |  |  |  |  | (.009) | | (.009) |
| Constant | 0.576*** | 0.535*** |  | 0.212 | 0.197 |  | 0.133 | | 0.130 |
|  | (.192) | (.192) |  | (.228) | (.226) |  | (.166) | | (.169) |

*Notes.* This table reports OLS coefficient estimates (robust standard errors corrected for clustering on the individual level in parentheses). For the risky choice tasks, the dependent variable is a dummy variable indicating whether participants chose the risky option. For the intertemporal choice task, the dependent variable is a dummy variable indicating whether they chose the immediate reward. “Pain” is a dummy for the pain condition. “Round” is a dummy for the second round of the tasks, i.e. the second time the participants performed the tasks. “Pain × Round” is the interaction between the pain condition and the task round, allowing the effect of pain to differ across the two task rounds. “Female” is a gender dummy. “Pain × Female” allows the effect of pain to differ between the two genders. “Age” is the participant’s age in years. “Ratio” is the ratio between the expected value of the risky option and the expected value of the safe option on each trial (standardized). “Delay” is the difference in days (1 or 5) between the immediate and the delayed reward in the intertemporal choice task.

* *p* < .10, ** *p* < .05, *** *p* < .01

**Table S4** Frequency (percent) of risky and impatient choices per trial in the first round of each task, as a function of condition (pain vs. control)

|  |  | Condition | |  | Chi-Square test |  |
| --- | --- | --- | --- | --- | --- | --- |
|  |  | Pain | Control |  | *p* |  |
| Risky gains | |  |  |  |  |  |
|  | 35 SEK vs. 100 SEK | 55 (98.2%) | 41 (82.0%) |  | .006 |  |
|  | 40 SEK vs. 100 SEK | 48 (85.7%) | 37 (75.5%) |  | .184 |  |
|  | 45 SEK vs. 100 SEK | 42 (75.0%) | 27 (57.4%) |  | .059 |  |
|  | 50 SEK vs. 100 SEK | 30 (52.6%) | 16 (32.0%) |  | .031 |  |
|  | 45 SEK vs. 90 SEK | 33 (60.0%) | 14 (29.2%) |  | .002 |  |
| Risky losses | |  |  |  |  |  |
|  | -35 SEK vs. -100 SEK | 5 (9.1%) | 5 (10.2%) |  | 1.00 |  |
|  | -40 SEK vs. -100 SEK | 13 (24.1%) | 5 (10.9%) |  | .087 |  |
|  | -45 SEK vs. -100 SEK | 16 (28.6%) | 12 (25.5%) |  | .730 |  |
|  | -50 SEK vs. -100 SEK | 35 (61.4%) | 26 (53.1%) |  | .386 |  |
|  | -45 SEK vs. -90 SEK | 32 (57.1%) | 23 (47.9%) |  | .347 |  |
| Intertemporal choice | |  |  |  |  |  |
|  | 50 SEK today vs. 60 SEK tomorrow | 3 (5.3%) | 4 (8.0%) |  | .703 |  |
|  | 50 SEK today vs. 60 in two days | 7 (12.3%) | 4 (8.2%) |  | .488 |  |
|  | 50 SEK tomorrow vs. 60 SEK in five days | 26 (45.6%) | 14 (28.0%) |  | .060 |  |
|  | 50 SEK tomorrow vs. 60 SEK in six days | 26 (46.4%) | 16 (32.0%) |  | .129 |  |

*Note.* When one or more cells had expected count less than five, Fisher’s exact test was used instead of Pearson chi-square test.

**Fig. S1** Distribution of difference scores (pain – control) for each task
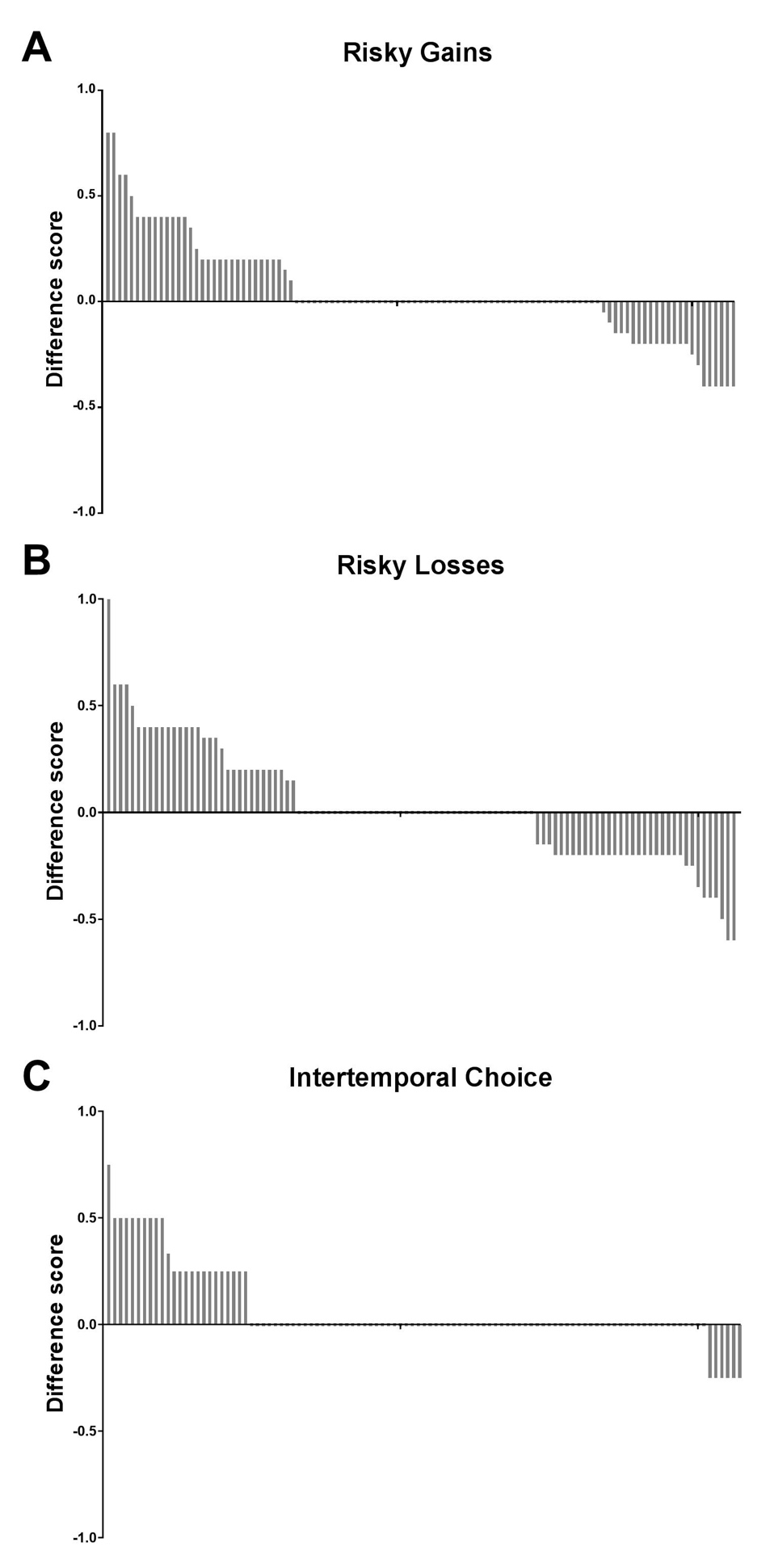


*Note*. Difference scores were computed for each task by subtracting the proportion of risky and impatient choices in the pain condition from the proportion of risky and impatient choices in the control condition. Each bar represents one participant.

**Experimental Instructions**

Welcome!

The experiment consists of 6 parts, each containing 5 questions.

In each question, you will make a decision about money. One of your answers will be randomly selected for actual payment in addition to the 100 SEK that you receive as a show-up fee.

It is important that you answer each question. If you don’t respond to one or more questions, and one of these is selected for actual payment, you will receive 0 SEK in total for your participation.

[Pain first condition] The thermode will be placed on your forearm in all 6 parts. In the first 3 parts you will receive pain stimulation. In the last 3 parts the thermode will be switched off.

[Control first condition] The thermode will be placed on your forearm in all 6 parts. In the first 3 the thermode will be switched off. In the last 3 parts you will receive pain stimulation.

Before each part of the experiment, you will receive instructions that you will need to read through carefully. If you have any questions, ask the experimenter before you begin each new part.

[New page]

INSTRUCTIONS PART 1

In the first 5 questions, you will choose between receiving a sum of money with certainty or to gamble. If you gamble, we will toss a coin and if it’s heads, you will receive an additional 90-100 SEK for your participation, while if it is tails, you will receive an additional 0 SEK for your participation. Thus, you have a 50% chance of winning an additional 90-100 SEK if you choose the coin toss.

You have 12 seconds to respond to each question. When 12 seconds have elapsed you will automatically move on to the next question.

Remember to answer each question.

Press ‘next’ when you are ready to begin. You will then see a timer that shows how much time is left before the first question is shown.

[New page]

Do you choose 35 SEK with certainty or to flip a coin for 100 SEK?

- 35 SEK with certainty
- Flip a coin for 100 SEK

*(Note: We used 35, 40, 45, and 50 SEK as the certain gain when the risky option was to flip a coin for 100 SEK, and 45 SEK as the certain gain when the risky option was to flip a coin for 90 SEK.)*

[New page]

INSTRUCTIONS PART 2

In the next 5 questions, you will choose between losing a sum of money with certainty or to gamble. If you gamble, we will toss a coin and if it is heads you will lose 0 SEK of your compensation for participating, while if it is tails you will lose 90-100 SEK of your compensation. Thus, you have a 50% chance of losing 90-100 SEK if you choose the coin toss.

You have 12 seconds to respond to each question. When 12 seconds have elapsed you will automatically move on to the next question.

Remember to answer each question.

Press ‘next’ when you are ready to begin. You will then see a timer that shows how much time is left before the first question is shown.

[New page]

Do you choose to lose 35 SEK with certainty or to flip a coin for a loss of 100 SEK?

- Lose 35 SEK with certainty
- Flip a coin for a loss of 100 SEK

*(Note: We used 35, 40, 45, and 50 SEK as the certain loss when the risky option was to flip a coin for a loss of 100 SEK, and 45 SEK as the certain loss when the risky option was to flip a coin for a loss of 90 SEK.)*

[New page]

INSTRUCTIONS PART 3

This part contains 5 questions. In the first 4 questions you will choose between two alternatives that mean that you will receive different sums of money at different points in time. In the last question you will face a hypothetical scenario.

You have 10 seconds to respond to the first 4 questions, and 20 seconds to respond to the last one. When the time is up you will automatically move on to the next question.

Remember to answer each question.

Press ‘next’ when you are ready to begin. You will then see a timer that shows how much time is left before the first question is shown.

[New page]

Do you want 50 SEK today or 60 SEK tomorrow?

- 50 SEK today
- 60 SEK tomorrow

*(Note: Choice options were 50 SEK today vs. 60 SEK tomorrow, 50 SEK today vs. 60 SEK in five days, 50 SEK tomorrow vs. 60 SEK in two days, and 50 SEK tomorrow vs. 60 SEK in six days.)*

[New page]

INSTRUCTIONS PART 4

In the next 5 questions, you will again choose between receiving a sum of money with certainty or to gamble. If you gamble, we will toss a coin and if it’s heads, you will receive an additional 90-100 SEK for your participation, while if it is tails, you will receive an additional 0 SEK for your participation. Thus, you have a 50% chance of winning an additional 90-100 SEK if you choose the coin toss.

You have 12 seconds to respond to each question. When 12 seconds have elapsed you will automatically move on to the next question.

Remember to answer each question.

Press ‘next’ when you are ready to begin. You will then see a timer that shows how much time is left before the first question is shown.

[New page]

Do you choose 35 SEK with certainty or to flip a coin for 100 SEK?

- 35 SEK with certainty
- Flip a coin for 100 SEK

*(Note: We used 35, 40, 45, and 50 SEK as the certain gain when the risky option was to flip a coin for 100 SEK, and 45 SEK as the certain gain when the risky option was to flip a coin for 90 SEK.)*

[New page]

INSTRUCTIONS PART 5

In the next 5 questions, you will choose between losing a sum of money with certainty or to gamble. If you gamble, we will toss a coin and if it is heads you will lose 0 SEK of your compensation for participating, while if it is tails you will lose 90-100 SEK of your compensation. Thus, you have a 50% chance of losing 90-100 SEK if you choose the coin toss.

You have 12 seconds to respond to each question. When 12 seconds have elapsed you will automatically move on to the next question.

Remember to answer each question.

Press ‘next’ when you are ready to begin. You will then see a timer that shows how much time is left before the first question is shown.

[New page]

Do you choose to lose 35 SEK with certainty or to flip a coin for a loss of 100 SEK?

- Lose 35 SEK with certainty
- Flip a coin for a loss of 100 SEK

*(Note: We used 35, 40, 45, and 50 SEK as the certain loss when the risky option was to flip a coin for a loss of 100 SEK, and 45 SEK as the certain loss when the risky option was to flip a coin for a loss of 90 SEK.)*

[New page]

INSTRUCTIONS PART 6

This part contains 5 questions. In the first 4 questions you will choose between two alternatives that mean that you will receive different sums of money at different points in time. In the last question you will face a hypothetical scenario.

You have 10 seconds to respond to the first 4 questions, and 20 seconds to respond to the last one. When the time is up you will automatically move on to the next question.

Remember to answer each question.

Press ‘next’ when you are ready to begin. You will then see a timer that shows how much time is left before the first question is shown.

[New page]

Do you want 50 SEK today or 60 SEK tomorrow?

- 50 SEK today
- 60 SEK tomorrow

*(Note: Choice options were 50 SEK today vs. 60 SEK tomorrow, 50 SEK today vs. 60 SEK in five days, 50 SEK tomorrow vs. 60 SEK in two days, and 50 SEK tomorrow vs. 60 SEK in six days.)*
